# Serum Proteome Analysis for Profiling Predictive Protein Markers Associated with the Severity of Skin Lesions Induced by Ionizing Radiation

**DOI:** 10.3390/proteomes1020040

**Published:** 2013-07-10

**Authors:** Thibault Chaze, Louis Hornez, Christophe Chambon, Iman Haddad, Joelle Vinh, Jean-Philippe Peyrat, Marc Benderitter, Olivier Guipaud

**Affiliations:** 1 Institut de Radioprotection et de Sûreté Nucléaire (IRSN), PRP-HOM, SRBE, LRTE, 31 avenue de la Division Leclerc, Fontenay-aux-Roses 92260, France; E-Mails: thibault.chaze@gmail.com (T.C.); marc.benderitter@irsn.fr (M.B.); 2 Laboratoire d’Oncologie Moléculaire Humaine, Centre Oscar Lambret, 3 rue Frédéric Combemale, BP 307, Lille 59020, France; E-Mails: L-Hornez@o-lambret.fr (L.H.); JP-Peyrat@o-lambret.fr (J.-P.P.); 3 PFEM, Composante Protéomique, UR370, INRA, Saint-Genès Champanelle 63322, France; E-Mail: christophe.chambon@clermont.inra.fr; 4 Spectrométrie de Masse Biologique et Protéomique, CNRS USR3149, ESPCI, 10 rue Vauquelin, Paris 75005, France; E-Mails: iman.haddad@espci.fr (I.H); joelle.vinh@espci.fr (J.V.)

**Keywords:** 2D-DIGE, biomarkers, cutaneous radiation syndrome, ionizing radiation, mass spectrometry, proteomics, radiotherapy, serum proteome, SELDI-TOF, skin

## Abstract

The finding of new diagnostic and prognostic markers of local radiation injury, and particularly of the cutaneous radiation syndrome, is crucial for its medical management, in the case of both accidental exposure and radiotherapy side effects. Especially, a fast high-throughput method is still needed for triage of people accidentally exposed to ionizing radiation. In this study, we investigated the impact of localized irradiation of the skin on the early alteration of the serum proteome of mice in an effort to discover markers associated with the exposure and severity of impending damage. Using two different large-scale quantitative proteomic approaches, 2D-DIGE-MS and SELDI-TOF-MS, we performed global analyses of serum proteins collected in the clinical latency phase (days 3 and 7) from non-irradiated and locally irradiated mice exposed to high doses of 20, 40 and 80 Gy which will develop respectively erythema, moist desquamation and necrosis. Unsupervised and supervised multivariate statistical analyses (principal component analysis, partial-least square discriminant analysis and Random Forest analysis) using 2D-DIGE quantitative protein data allowed us to discriminate early between non-irradiated and irradiated animals, and between uninjured/slightly injured animals and animals that will develop severe lesions. On the other hand, despite a high number of animal replicates, PLS-DA and Random Forest analyses of SELDI-TOF-MS data failed to reveal sets of MS peaks able to discriminate between the different groups of animals. Our results show that, unlike SELDI-TOF-MS, the 2D-DIGE approach remains a powerful and promising method for the discovery of sets of proteins that could be used for the development of clinical tests for triage and the prognosis of the severity of radiation-induced skin lesions. We propose a list of 15 proteins which constitutes a set of candidate proteins for triage and prognosis of skin lesion outcomes.

## 1. Introduction

Damage to the skin induced by ionizing radiation is complex and leads to a defined range of specific reactions that frequently turn into a pathophysiological process: the cutaneous radiation syndrome (CRS) [1,2,3]. In the case of both accidental exposure and radiotherapy, high doses of ionizing radiation induce reactions that arise from days to years after exposure to radiation doses greater than 20 Gy, and which can extend to different grades of severity, as erythema, dry and moist desquamation, ulceration and necrosis characterized by several unpredictable waves of recurrences [4]. Reports on several radiation accidents have clearly shown that skin damage can determine the prognosis and outcome of the whole-body reaction [5]. Additionally, the probability of an individual being exposed to nuclear weapons or dispersed nuclear material has significantly increased during the past two decades [6], highlighting that it is crucial to manage the consequences of radiation diseases, including those linked to local irradiation, and to predict as early as possible the severity of the impending lesion.

The extent of radiation-induced reactions does not exclusively depend on the dose received. Especially the severity of skin lesions induced by local exposure will significantly depend on the surface area and volume exposed to radiation. The dose received can be estimated by advanced techniques focused on different cytogenetic, genetic, physical and immunohistochemical parameters [7]. Nevertheless, these techniques are more suitable for whole- and partial-body irradiation than for local radiation exposure, require considerable expertise and are generally time-consuming. Also, whereas a radiation burn is possible to diagnose, it is hard to predict its outcome, particularly when the lesion is localized. Among available therapeutic strategies for local injury, stem cell-based therapies are promising [8,9,10,11,12]. However, the use of such therapies needs to be anticipated as early as possible in order to allow enough time to isolate and amplify cells before treatment, highlighting the need for new tools for better prediction of the severity of localized injuries. Since the METREPOL (medical treatment protocols for radiation accident victims) approach is based on clinical symptoms [13,14], there is a need to develop other markers to reinforce diagnosis strategies, particularly when the time of exposure is not known or when the accident is disclosed beyond the first 48 h. For example, following a radioactive source accident, the victim can be severely exposed locally but it is currently impossible to predict the outcome of the cutaneous lesion (*i.e.*, necrosis or not). In such cases, it is critical to develop biomarkers which predict the severity of the lesion. These markers would clearly help clinicians to make the right decisions in a timely manner and to choose the most suitable therapeutic strategy.

As organisms respond to irradiation by altering the expression and/or the post-translational modifications of some proteins in cells, tissues and organic fluids, as serum, plasma or urine, it is conceivable that protein expression profiling can be used to define protein or, potentially much better, a set of protein expression changes that differentiates between irradiated and non-irradiated individuals, or that differentiates early between detrimental and harmless impending injuries in the case of restricted exposures. Applied to radiation biology, omics are interesting approaches suitable for discovering biomarker candidates and describing pathways involved in the response to ionizing radiation, giving rise to a new understanding of pathophysiological modifications [15,16,17]. Using two-dimensional differential in-gel electrophoresis (2D-DIGE) coupled with mass spectrometry, we showed that serum proteome content is deeply altered from one day to one month following exposure of the skin to a high dose of ionizing radiation [18]. On the other hand, using a large-scale quantitative glycomic approach based on a mass spectrometry methodology, we observed changes in post-translational modifications of serum proteins, *i.e.*, increases in multiantennary N-glycans and outer branch fucosylation and sialylation, following radiation exposure of the skin [19]. Among proteomics technologies, SELDI-TOF-MS has been particularly promising for fast protein profiling of biological samples with clinical applications because it allows high-throughput intact protein analysis of complex biological samples with limited preprocessing steps [20,21]. It has been used to identify protein patterns related to various stages and types of solid tumors both in tissue and serum [20,22,23,24,25,26,27,28,29]. Interestingly, the potential of SELDI-TOF-MS has been explored in patients with cancer before and during radiotherapy in an effort to discover clinical biomarkers of radiation exposure [30]. Computer-based analyses of the SELDI protein spectrums could distinguish unexposed from radiation-exposed patients using various classifier models. The method also showed an ability to distinguish high from low dose-volume levels of exposure. One could thus conceive that SELDI-TOF-MS may be used as a mobile clinical apparatus for triage of thousands of irradiated people.

In this paper, we used global proteomics approaches to look for serum proteins altered in expression during the early post-irradiation phase. The two proteomic methodologies 2D-DIGE-MS and SELDI-TOF-MS, in association with unsupervised and supervised multivariate statistical analyses (PCA, Random Forest and PLS-DA), were used to establish protein profiles that could be relevant for early discrimination between groups that will develop different skin lesions. We show that, unlike SELDI-TOF-MS, the 2D-DIGE approach remains a powerful method for the discovery of sets of proteins that could be used for the development of clinical tests for triage and the prediction of radiation-induced skin lesion severities.

## 2. Experimental

### 2.1. Animals

Male mice (BALB/c, 8 weeks old, Janvier) were used in this study. Experimental procedures were approved by the animal care committee of the French Institute for Radiological Protection and Nuclear Safety (institutional review board (IRB) approval number P07-12) and were conducted according to the French regulations for animal experimentation (Ministry of Agriculture, Act No. 2001-464, May 29, 2001). Mice were housed four per cage and received rodent chow and water *ad libitum*.

### 2.2. Dorsal Skin Irradiation of Mice

**Figure 1:**
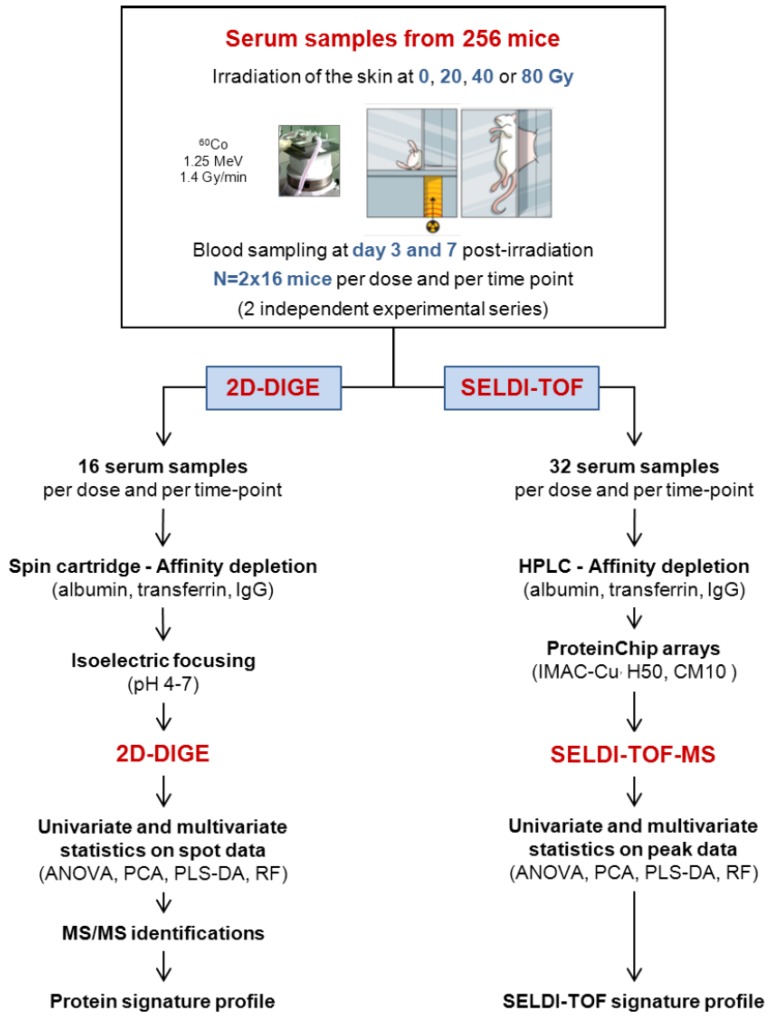
Experimental strategy for the discovery of markers of the cutaneous radiation syndrome using proteomics approaches.

The irradiation protocol used in this study is mostly the same as previously published [18] (Figure 1). Mice were irradiated at different doses (0, 20, 40, and 80 Gy, 32 mice per dose and per blood collection time point, two independent experimental series of n = 16 mice each). Two days before irradiation, hair from the entire back of the mouse was removed using an electric shaver. Mice were anesthetized by spontaneous inhalation of isoflurane–N_2_O gas (Forene, Abbott GmbH) and irradiated using 1.25 MeV γ-rays of a collimated ^60^Co source (dose rate of 1.4 Gy/min). The dorsal skin was gently stretched and maintained on a 4 mm-thick polymethyl methacrylate (PMMA) plate with two suturing lines (skin surface irradiated: 12 cm^2^, *i.e.*, 20% of the total skin surface). The reference dose rate was established with an ionization chamber (PTW) in realistic irradiation conditions in air on the PMMA plate with a tissue equivalent phantom at mouse body position to generate back-scattered radiation from the mouse bodies. The PTW ionization chamber of 0.125 cm^3^ was calibrated in terms of tissue kerma at the IRSN reference ^60^Co facility (accredited metrological unit SMH No. 2-1612 – COFRAC accreditation, France).

### 2.3. Mouse Skin Lesion Scoring System

Lesions to the skin were scored daily in a non-blinded manner using a single scale, as previously described [18]. Briefly, the lesions were scored as follows: 0, normal; 0.5–1, slight to intense erythema; 1–1.5, dry desquamation; 1.5–2.5, moist desquamation (less than 50% of the irradiated area); 2.5–3, a few spots of necrosis in addition to 50%–100% of moist desquamation; 3–3.5, several zones of necrosis in addition to 100% of moist desquamation; 3.5, necrosis of the entire irradiated area.

### 2.4. Mouse Biological Sampling and Affinity Depletion of High Abundance Proteins

Blood (100 µL per animal) was collected from the orbital sinus of anesthetized mice (spontaneous inhalation of isoflurane–N_2_O gas) 3 and 7 days after irradiation. After 30 min at room temperature, blood was centrifuged at 1,000 ×*g* for 10 min at 4 °C and the supernatant was collected. Serum was then stored at −80 °C. For 2D-DIGE experiments, serum samples (20 µL each, 16 mice per dose and per time point) were processed using a mouse Multiple Affinity Removal System Spin Cartridge (Agilent Technologies), which selectively removed mouse albumin, IgG and transferrin proteins, as previously described [18]. For SELDI-TOF experiments, serum samples (55 µL each, 32 mice per dose and per time point) were processed using the Alliance HPLC system (Waters) with a mouse Multiple Affinity Removal System Liquid Chromatography Column (4.6 × 50 mm, Agilent Technologies), which selectively removed mouse albumin, IgG and transferrin proteins. A low abundance protein fraction was collected and ten-fold concentrated using a 3 kDa molecular weight cut-off spin concentrator (Amicon Ultra 0.5, Millipore) by centrifugation at 14,000 ×*g* for 45 min at 20 °C. Two hundred μg of each sample was first diluted in the dialysis buffer (7 M urea, 2 M thiourea, 0.5% CHAPS, 18 mM DTT) to reach a final concentration of 2 μg/μL, and then dialyzed overnight at room temperature with mixing against 100 volumes of the dialysis buffer using a dialysis tube (Mini Dialysis Kit, 1 kDa molecular weight cut-off, GE Healthcare). Protein concentrations were determined by means of the Bradford assay (Bio-Rad). The quality of the depletion of each sample was checked by the migration of 5 µg of proteins on SDS-PAGE (MiniProtean system, Bio-Rad).

### 2.5. 2D-DIGE Experimental Design

A comparative 2D-DIGE approach was performed with depleted mouse serum proteins. High abundance-depleted serum proteins (50 µg) were minimally labeled with 400 pmol of Cy2, Cy3 or Cy5 DIGE fluors (GE Healthcare) as described previously [18]. Samples consisted of depleted serum from individual control mice (16 samples from 2 independent experiments) and mice irradiated at 20 Gy (16 samples from 2 independent experiments), 40 Gy (16 samples from 2 independent experiments) and 80 Gy (16 samples from 2 independent experiments). A total of 64 SDS-PAGE were performed, each gel containing two different samples (Cy3- and Cy5-labeled) and an internal standard sample (Cy2-labeled). A dye-swapping scheme was used so that the 16 samples from any condition were labeled half by Cy3 and half by Cy5 to avoid any specific dye labeling artifacts that might occur.

### 2.6. Two-Dimensional Gel Electrophoresis and Imaging

Protein samples labeled with Cy2, Cy3 and Cy5 dyes were mixed and diluted with rehydration buffer to 450 μL as previously described [18]. One hundred and fifty μg (combination of the three labeled protein samples) of protein was applied to 24-cm-long immobilized pH 4–7 gradients (IPG) strips (GE Healthcare) by the passive rehydration technique for 24 h. First-dimension isoelectric focusing (IEF) was performed using an IPGPhor 3 electrophoresis unit (GE Healthcare) cooled to 18 °C for a total of 55 kVh. After IEF, strips were equilibrated and placed on top of hand-cast SDS-containing 10% polyacrylamide gels and SDS-PAGE was then carried out at 0.5 W per gel for 1 h followed by 1 W per gel for 16 h (Ettan DALT 12 system, GE Healthcare) as previously described [18]. After electrophoresis, gels were scanned directly between glass plates using a Typhoon 9400 imager (GE Healthcare). Preparative gels (400 µg of depleted serum proteins) for spot excision were fixed either in 30% ethanol and 2% orthophosphoric acid 85% for 2 h prior to colloidal Coomassie brilliant blue G-250 staining or in 30% ethanol and 10% acetic acid for 2 h prior to silver staining. The gels were kept in a 1% acetic acid solution until spot excision for MS analysis. Images stained with Coomassie brilliant blue and silver nitrate were acquired on a molecular imager GS-800 calibrated densitometer (Bio-Rad).

### 2.7. 2D-DIGE Data Evaluation and Statistical Analysis

2D-DIGE scanned gels were analyzed with Progenesis SameSpots software version 4.1 (NonLinear Dynamics). The differential in-gel analysis module was used to quantitatively compare the normalized volume ratio of each individual protein spot-feature from a Cy3- or Cy5-labeled sample on a given gel relative to the Cy2-signal from the pooled sample standard corresponding to the same spot-feature. Within each gel, the co-resolved fluorescent signals from each protein are co-detected by the software and abundance measurements are made directly to the internal standard without interference from gel-to-gel variation. One-way analysis of variance (ANOVA) was performed with Progenesis SameSpots software and was used to calculate significant differences in relative abundances of proteins from the different groups of mice. Principal component analysis (PCA) of 2D-DIGE quantitative data was performed using the statistical module of Progenesis SameSpots software and was used to interpret relationships between experimental groups.

### 2.8. SELDI-TOF Experiments

A comparative SELDI-TOF approach was used with depleted mouse serum proteins. Samples consisted of high abundance-depleted serum proteins (70 µg) from individual control mice (32 samples from two independent experiments) and mice irradiated at 20 Gy (32 samples from two independent experiments), 40 Gy (32 samples from two independent experiments) and 80 Gy (32 samples from two independent experiments). Three types of ProteinChips (Bio-Rad) with a surface chemistry of hydrophobic (H50), cationic (CM10) and metal affinity (IMAC-Cu) were tested to determine which might provide the best mouse depleted serum profiles (data not shown). The two chromatographic surfaces CM10 and IMAC-Cu were further selected for serum profiling. CM10 were precharged with 100 mM sodium acetate pH 4.0. IMAC-Cu were precharged for activation with 100 mM CuSO_4_ for 10 min and neutralized twice with phosphate-buffer saline (PBS) pH 7.4, according to the manufacturer’s instructions (Bio-Rad). Seventy µg of depleted serum per sample was incubated for 30 min with gentle shaking on the ProteinChips using a 96-well bioprocessor (Bio-Rad) and the arrays were washed twice with 200 μL of adequate binding buffer for 5 min, followed by two quick rinses with 200 μL of deionized water. After air-drying, 1 μL of saturated sinapinic acid (5 mg dissolved in 400 μL of acetonitrile/ trifluoroacetic acid (50%/0.5%)) was applied twice to each spot, allowing the array surface to air-dry for 10 min between each application. Proteins bound to the ProteinChip arrays were detected with the ProteinChip System Series 4000 (Bio-Rad). Time of flight spectra were generated by averaging 500 laser shots collected at a laser intensity of 2,500 with a focus mass of 7,000 (for 1,800–10,000 Da proteins), at a laser intensity of 2,500 with a focus mass of 16,000 (for 1,800–20,000 Da proteins), and finally at a laser intensity of 5,000 with a focus mass of 40,000 (for 20,000–150,000 Da proteins). External mass calibration was performed using the All-In-One Peptide molecular mass standard. Spectral analyses (peak detection, mass calibration, baseline subtraction and total ion current normalization) were performed using Ciphergen ProteinChip Data Manager DE Software 3.0 (Bio-Rad). Auto-detect peaks to cluster was done after normalization (on the total ion current) with a signal-to-noise ratio (S/N) < 2.5% and 50% minimum peaks of all for the first pass. For cluster completion, the S/N ratio was 3%, with a minimum peak threshold of 10% and 0.3% of mass for the cluster window. On each array, a standard serum, corresponding to a pool of all analyzed serums, was spotted to evaluate the variability. Reproducibility was estimated using this pool by the mean of the coefficient of variation, for all the detected peaks at each laser intensity and for each type of ProteinChip, and ranged from 10% to 20%.

### 2.9. Multivariate Statistical Analysis

Best candidates for triage and prognosis of radiation-induced skin burns were searched for with a partial least square discriminant analysis (PLS-DA) and a Random Forest method applied to 2D-DIGE and SELDI-TOF data (Anastats, www.anastats.fr). 

For PLS-DA, a recursive partitioning was performed for every analysis by dividing the total sample size into a learning sample (70% of the sample size) and a test sample (30% of the sample size). The composition of these two groups was randomly sampled. At the end of every analysis, we recorded the proportion of well classified individuals, for the learning and the test samples, in the confusion matrix. Variables were centered and reduced. For each category of discrimination, 10 different PLS-DAs were repeated on data. Following each of these 10 analyses we kept 30 variables having the highest importance for the discrimination. Among the 10 series of 30 variables so selected we qualified as “good” variables selected by the PLS-DA those that were present 8 to 10 times on 10 drawings. They were generally from three to eight different variables. The most important variables chosen by this method were then sorted by a linear discriminant analysis (LDA) using a cross-validation and a stepwise procedure. This model was then used for the calculation of a confusion matrix and a ROC curve. A linear discriminating analysis using the same variables then allowed a graphic representation of the quality of the discrimination on two histograms along the discriminating axis.

For Random Forest, every elementary analysis generated 1,000 successive segmentations, each using 20 variables chosen randomly and a bootstrap sample of the same length as the total number of individuals. At the end of every elementary analysis, all the variables were sorted in order of importance in the discrimination. The 10 best variables obtained at the end of 1,000 segmentations were recorded. These elementary analyses were repeated 30 times, the random sampling being reset on each iteration. We then listed the 10 best variables, which were retained for each elementary analysis, and counted the number of times they were in the 10 most important variables for the 30 iterations. Between 4 and 10 important variables were thus selected. Elementary statistics and a non-parametric test using the method of permutations (Monte Carlo approximation of the exact probability) were performed to compare the groups. *p*-values were corrected by the Benjamini and Yekutieli method (control of the false discovery rate (FDR) for multiple comparisons) [31]. The control of the FDR used here the totality of the variables, and not only the important selected variables. A logistic regression among the important variables selected by the 30 repetitions of the Random Forest method was then used to search for the best variables. The best model of regression was then calculated by a stepwise method using the Akaike information criterion (AIC). Variables retained by this method were considered as the best variables. A graphic representation in Tukey diagrams was plotted (box plots). This model was then used for the calculation of a confusion matrix and a ROC curve. A linear discriminating analysis using the same variables then allowed a graphic representation of the quality of the discrimination on two histograms along the discriminating axis (data not shown). The ROC curve and AUC obtained by the best set of candidates for the discrimination of groups were given. The “caret” R-package was used for the PLS-DA analysis and the Random Forest method. The XLSTAT software was used for LDA and the ROC curve.

### 2.10. Protein Identification

Spots of interest were manually excised from Coomassie brilliant blue or silver stained 2D gels. The same spots from two or three preparative gels were pooled and subjected to in-gel tryptic digestion, as previously described [32]. Two different methodologies were used for identification of proteins.

In the first methodology, the peptide hydrolysates were analyzed by using a DE-PRO MALDI-TOF mass spectrometer (Applied Biosystems) in positive ion reflectron mode. The samples (1 μL) were deposited on the MALDI target with 1 μL of α-cyano-4-hydroxycinnamic acid matrix dissolved in a 1:1 (v/v) ACN/H_2_O, with 0.1% TFA (v/v) solution. The internal calibration of spectra was performed by using the autolysis peaks of trypsin and noise filtration. The resulting peak lists were generated by the DataExplorer 4.0 software (Applied Biosystems). Peptide mass fingerprints were searched by querying the SwissProt database restricted to the sequences of mouse (01/2010, 16215 sequences), with the search engine MASCOT v2.1. The parameters used for the search in the database were the following: cysteine carbamidomethylation and methionine oxidation as variable modifications, one miscleavage of trypsin, precursor error tolerance of 30 ppm. The MASCOT probabilistic scores were used to validate protein identifications (significant score at *p* < 0.01). When MALDI-TOF analyses were unsuccessful, the samples were analyzed with an LCQ Deca IonTrap mass spectrometer (ThermoFisher) coupled with a nanoHPLC (Dionex) by using the method described previously [33]. Raw data were processed with Bioworks 3.1 (ThermoFisher) and queries were performed using the same database as that used for PMF analysis Peptides were validated when their Mascot score was significant at *p* < 0.05. The identification of a protein was accepted with at least two validated peptides.

In the second methodology, peptide mixtures were analyzed using a 4800 Proteomics analyzer MALDI-TOF/TOF mass spectrometer (Applied Biosystems) in positive ion reflectron mode. MS/MS data were obtained using air as collision gas at 2 keV collision energy. Samples (0.3 mL) were deposited with 0.6 mL of a CHCA matrix dissolved in a 6:4 v/v ACN/H2O, with 0.1% TFA v/v solution. An external plate model calibration was achieved with a standard peptide mix (Proteomix Peptide mix4, LaserBioLabs) containing: bradykinin fragment 1–5 (573.3150 Da), human angiotensin II (1046.5424 Da), neurotensin (1672.9176 Da), ACTH fragment 18–39 (2464.1989), and oxidized insulin B chain (3494.6514 Da). After MS acquisition, the top 10 most intense peptides per spot were selected automatically for the MS/MS analysis. The search engine MASCOT v2.1 was used to search data *versus* the Uniprot-SwissProt database (available from EBI), and the GPS Explorer™ software version 2.0 (Applied Biosystems) was used to create and search files with the MASCOT search for peptide and protein identification. The identification was restricted to tryptic peptides in mammals or humans, with cysteine carbamidomethylation and methionine oxidation as variable modifications, two miscleavages, precursor error tolerance of 50 ppm and MS/MS fragment error tolerance of 0.15 Da. Individual ions scores greater than 30 were set as the acceptance threshold. Peptide mass fingerprints were also manually searched using the Profound Web-based database with cysteine carbamidomethylation and methionine oxidation as partial modifications, 0–1 miscleavage, and monoisotopic mass tolerance from 0.03 to 0.4 Da. The search results were evaluated by considering a probability value of 1, the estimated Z-parameter, the peptide coverage, the pattern of coverage and the correspondence of predicted and experimental pI and molecular mass. When MALDI-TOF/TOF analyses were unsuccessful, the samples were analyzed with LC-MS/MS. Five μL of peptides diluted 1:5 in water (5% of the total amount of material) was purified on a capillary reversed phase column (nano C18 Acclaim PepMap100, 75 μm i.d. × 15 cm length column, Dionex), at a constant flow rate of 220 nL/min, with a gradient 2% to 40% B in 90 min; buffer A: water/ACN/AF 98:2:0.1 (v/v/v); buffer B water/ACN/FA 10:90:0.1 (v:v:v). The MS analysis was performed on an FT-ICR mass spectrometer (LTQ-FT Ultra, ThermoFisher Scientific) with the top 7 acquisition method: MS resolution 60,000, between 500 and 2000 Th, followed by 7 MS/MS (LTQ) on the 7 most intense peaks, with a dynamic exclusion for 90 s of the previously fragmented precursors. The raw data were processed using Xcalibur 2.0.7 software. The database search was done using the Mascot search engine (Matrix Science Mascot 2.3) in the *Mus musculus* databank. The following parameters were used: up to 2 miss cleavages; MS tolerance 10 ppm; MSMS tolerance 1 Da; full tryptic peptides; partial modifications such as carbamidomethylation (C), oxidation (M, H, W), phosphorylation (Y); MudPIT scoring; significance threshold *p* < 0.05. Protein identifications were validated only if they satisfied these three requirements: (i) their score was significant (*p* < 0.05) with cut-off criteria; (ii) they were identified with one peptide with a score above 47 or two peptides with a score above 30; and (iii) they were identified in at least two out of the three runs. Proteins identified by a set or subset of peptides used for identification of another protein were not taken into account. Five supplemental spots were analyzed by LC-MS/MS performed on a Q Exactive mass spectrometer (Thermo Scientific) with the top 10 acquisition method: MS resolution 70,000, between 400 and 2,000 Th, followed by 10 MS/MS (Orbitrap), resolution 7,500, on the 10 most intense peaks, with a dynamic exclusion for 30 s of the previously fragmented precursors. The raw data were processed using Proteome Discoverer 1.3 software. The database search was done using Mascot search engine (Matrix Science Mascot 2.4) on the *Mus musculus* databank. The following parameters were used: up to 2 miss cleavages; MS tolerance 5 ppm; MSMS tolerance 0.5 Da; full tryptic peptides; partial modifications such as carbamidomethylation (C), oxydation (M, H, W), Phosphorylation (Y); MudPIT scoring; significance threshold *p* < 0.05. Protein identifications were validated only if they satisfied these three requirements: (i) their score was significant (*p* < 0.05) with cut-off criteria; (ii) they were identified with one peptide with a score above 47 or two peptides with score above 30; and (iii) they were identified in at least two out of the three runs. All data were processed and validated by *myProMS*, a web server for management and validation of mass spectrometry-based proteomic data [34].

### 2.11. Pathway Analysis of Differentially Expressed Proteins

Pathway analysis and ontology and sub-network enrichment analyses were performed using Pathway Studio 9.0 along with ResNet 9.0 from Elsevier, the database of functional relationships and pathways of mammalian proteins (www.elsevier.com/pathway-studio). SwissProt identifiers of differentially expressed proteins were imported into the Pathway Studio. This web-based database consists of millions of individually modeled relationships between proteins, genes, complexes, cells, tissues, drugs and diseases [35]. The data input was queried against the Pathway Studio knowledge base for biological interactions. Proteins mapped to the knowledge base were used to build protein networks. An interaction network was added that included the upstream regulators.

## 3. Results and Discussion

### 3.1. Experimental Strategy

The experimental strategy is shown in Figure 1. The experimental model of the CRS consists in selectively irradiating the dorsal skin of BALB/c mice, as previously described [18]. 

We used this model by irradiating mice at 20, 40, and 80 Gy to induce, respectively, erythema, dry and moist desquamation, and necrosis of the skin in the course of the development of the lesion. Blood samples were collected from 256 mice during the clinical latency phase, *i.e.*, before the appearance of the macroscopic lesions, at day 3 and day 7 post-irradiation. Samples were obtained from two different experimental series each including 16 mice per time point and per dose of irradiation, which made a total of 32 serum samples per time point and per dose. We chose to analyze the data from the two experiments together rather than analyzing the datasets separately, even if it could reduce the list of candidate biomarkers, because this method could abolish a potential batching effect in animal source and animal handling. The use of a large number of mice was absolutely required for running multivariate statistical tests such as PLS-DA and/or Random Forest since these statistical analyses need to separate the samples into a learning sample (70% of the sample size) and a test sample (30% of the sample size). Because 2D-DIGE is time-consuming and relatively expensive, only 16 samples per time point and per dose were analyzed, while the 32 samples per time point and per dose were assessed using three different chemical ProteinChips surfaces for SELDI-TOF experiments. Serum samples were depleted from the three high abundance proteins albumin, transferrin and IgG before proteomics analysis. Depletion allowed the detection and the quantification of many more protein spots, as previously shown and discussed [18], and peaks (data not shown). Univariate (ANOVA) and multivariate statistics (PCA, PLS-DA and Random Forest) were then used to explore the datasets. Differentially expressed proteins detected by 2D-DIGE were further identified by mass spectrometry.

### 3.2. Animal Model and Scoring of Irradiated Mouse Skin Lesions

**Figure 2:**
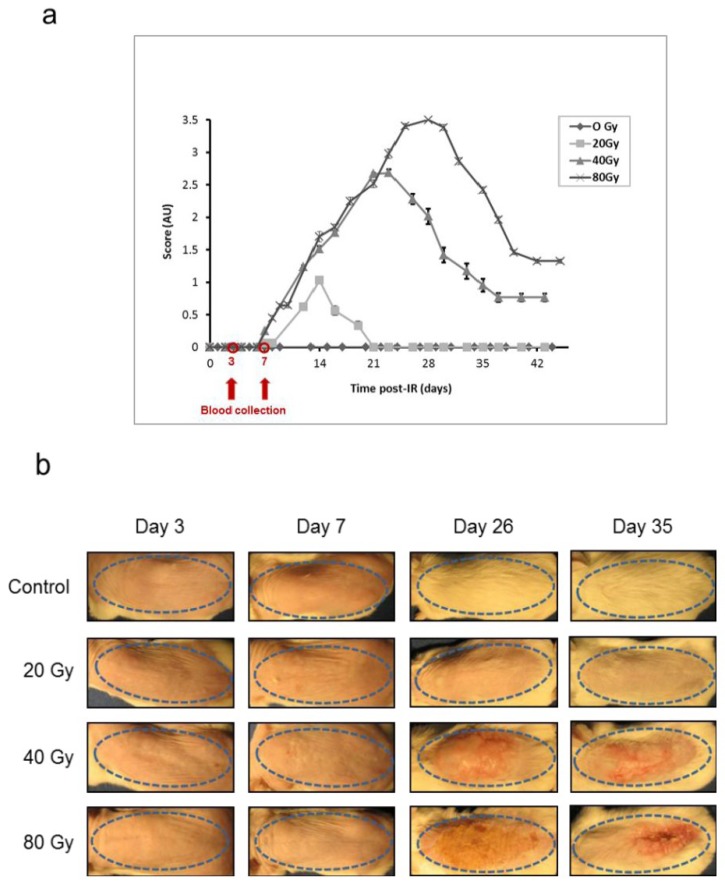
Animal model for the study of the cutaneous radiation syndrome (**a**) Progression of skin lesions in mice following irradiation at different doses (n = 16 per group. Mean ± SEM in arbitrary units, AU); (**b**) Representative animals 3, 7, 26 and 35 days following irradiation (Control: sham-irradiated). The skin area of irradiation is shown by a blue dotted ellipse.

We used the experimental model of the CRS we previously established by selectively irradiating the dorsal skin of BALB/c mice [18,19,36] at 20, 40, and 80 Gy to induce respectively erythema, dry and moist desquamation and necrosis of the skin in the course of the development of the lesion. As previously observed, lesions developed from the seventh day post-irradiation whatever the dose of ionizing radiation (Figure 2a). The lesions evolved from erythema (score 0.5–1) to necrosis (score 3–3.5) as a function of time and dose of ionizing radiation, reaching a peak around 2 weeks (20 Gy), 3 weeks (40 Gy) and 4 weeks (80 Gy) following exposure. Slow wound healing took place over different times depending on the exposure dose. During the first week post-irradiation, the animals presented no lesions whatever the dose of irradiation, while the lesions evolved toward different maximum intensities afterwards (Figure 2b). This latency phase was chosen to study mouse proteome changes at day 3 and day 7 post-irradiation.

### 3.3. Differential 2D-DIGE and SELDI-TOF Analyses of Serum Proteins

**Figure 3:**
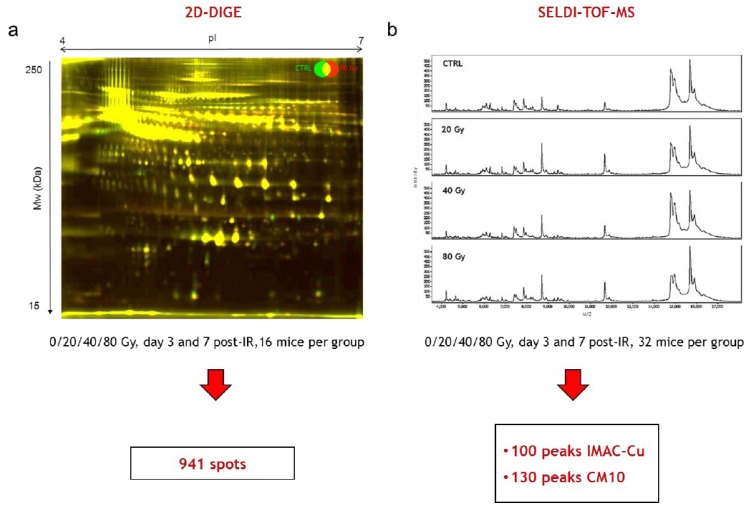
Differential 2D-DIGE and SELDI-TOF analyses of serum proteins (**a**) Representative 2D-DIGE gel. Merged 2D-DIGE image of proteins from a mouse irradiated at 80 Gy (red) and a control mouse (green) 7 days after irradiation; (**b**) Representative SELDI-TOF serum protein profiles 7 days after irradiation on IMAC-Cu from a control mouse and mice irradiated at 20, 40 and 80 Gy.

Sixteen samples per time point and per dose, from a total number of 128 mice from the two independent experimental series, were analyzed on a total of 64 2D-DIGE gels. An example of a merged 2D-DIGE image is shown in Figure 3a. In-gel image analysis using the Progenesis SameSpots software allowed the positioning and the quantification of 941 spots on gels at any time post-irradiation and any dose of irradiation. This number of spots is similar to the numbers we obtained in our previous work using the same methodology and the same model of irradiation [18,19]. It should be noted that at this stage of the analysis, contrary to what we observed in our previous work for later times (14, 21 and 33 days post-irradiation), no differentially expressed spot could be clearly and reproducibly identified by simple visual examination of gels. 

For SELDI-TOF experiments, the 32 samples per time point and per dose from a total of 256 mice from the two independent experimental series were assessed using the three types of ProteinChips H50, CM10 and IMAC-Cu. Analysis of the serum samples on H50 arrays led to results with too high a variability (data not shown). On the other hand, CM10 and IMAC-Cu arrays provided reproducible profiles showing respectively around 130 and 100 peaks detected and analyzed by the Ciphergen ProteinChip Data Manager DE software 3.0. Examples of SELDI-TOF serum protein profiles are shown in Figure 3b. At this stage of the analysis, no protein peak differentially expressed after irradiation could be clearly identified visually.

#### 3.3.1. SELDI-TOF Analysis

**Figure 4:**
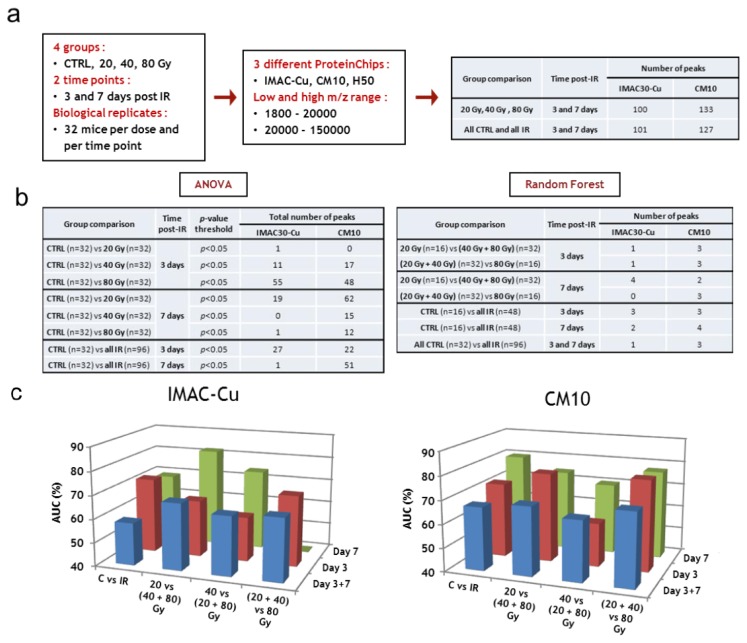
SELDI-TOF analysis. (**a**) Workflow of protein peak detection; (**b**) Statistical tests, group comparisons and number of selected peaks; (**c**) Supervised statistical analysis of SELDI-TOF dataset. CTRL, sham-irradiated. IR, irradiated.

Low and high m/z ranges of serum proteins were analyzed by SELDI-TOF on the three different types of ProteinChips (Figure 4a). Two different group comparisons were performed for each type of ProteinChip to look for common peaks: (i) a comparison of samples from all non-irradiated mice with samples of all irradiated mice, leading to the detection and quantification of 101 proteins for IMAC-Cu and 127 proteins for CM10; (ii) a comparison of the groups according to the dose of irradiation, with the exception of control samples, leading to the detection and quantification of 100 proteins for IMAC-Cu and 133 proteins for the CM10. The mass lists and their corresponding peak intensities (raw data) are given in the Supplemental Table S1.

The data obtained with IMAC-Cu and CM10 arrays were then analyzed by univariate (ANOVA) and multivariate (PLS-DA and Random Forest) statistics to look for the best candidates for triage and prognosis of radiation-induced skin burns. ANOVA with a *p*-value threshold of 0.05 was able to highlight a number of protein candidates for assessing either dose or exposure (Figure 4b). However, the variations between the means were very low and the protein candidates differed from one group comparison to another, suggesting that the differences were due to random variations. Furthermore, *p*-values were not adjusted to control the FDR for multiple testing at this stage of the analysis because they were relatively high. Since we had a large number of samples (32 per group), we then decided to mine the data using supervised multivariate statistical analyses. While the Random Forest method allowed the sorting of important variables that play an effective role in the discrimination of groups, the PLS-DA method was unable to do this with these data. The reason is that, most of the time, the differences in treatments affect some individuals and have little effect on the majority of subjects. However, since a few individuals were enough to make pure segments, this allowed characterization of important variables with the Random Forest method. Between 4 and 10 important variables were thus selected. Figure 4b lists the number of variables that were selected by this method for each comparison. The best sets of candidates for the discrimination of groups were used to build and calculate confusion matrices, ROC curves and AUCs. Figure 4c displays the AUC for every group comparison with either IMAC-Cu or CM10. It shows that AUCs were mostly less than 70%, and always less than 80%, indicating poor discrimination between groups.

The discriminations made in this study were altogether not very marked. This was mainly due to high inter-individual variability in response to treatments. We frequently noted that some individuals were clearly separated between the various groups, without there being large differences for the whole group. The results obtained with CM10 were generally a little better than with the IMAC. This was particularly marked for the comparisons of the groups on day 3. In conclusion, no important variables were found for the discrimination of groups in the SELDI-TOF experiments. An explanation was that the various treatments affect only some individuals and have little effect on most subjects. As a consequence, because various treatments affect only some individuals, important variables could be characterized, but discrimination remained poor. In this context, the use of SELDI-TOF for high-throughput triage appears compromised.

#### 3.3.2. Differential 2D-DIGE Analysis and MS Identifications

**Figure 5:**
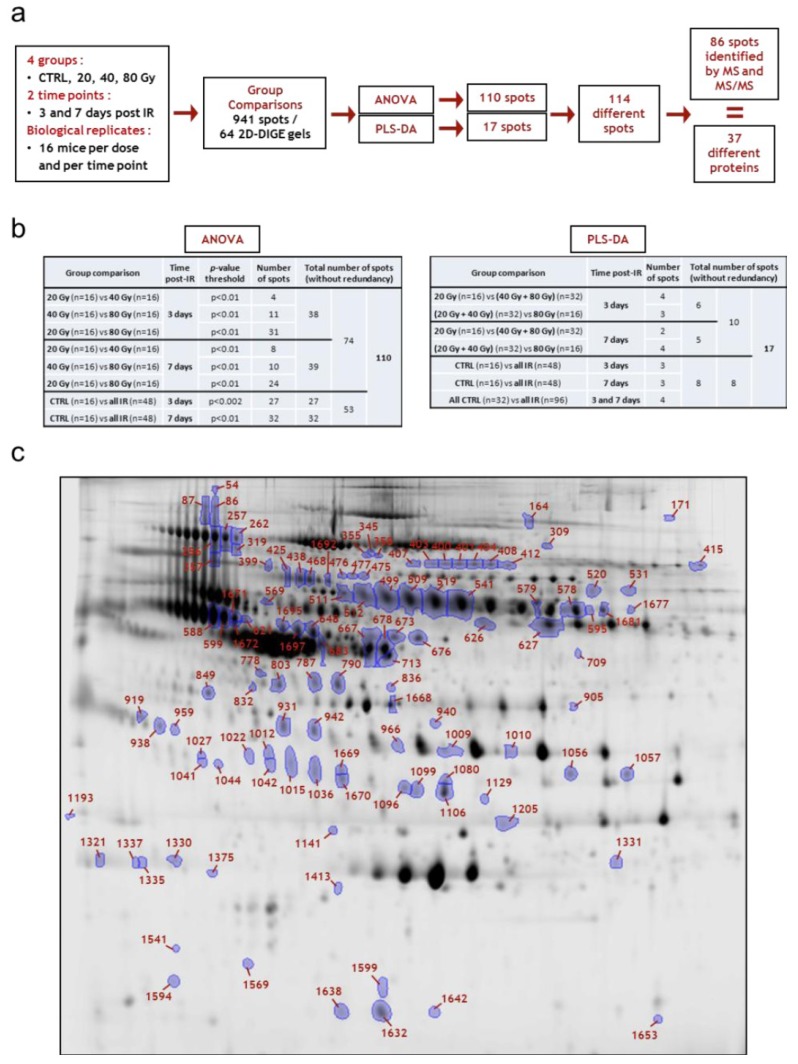
Differential 2D-DIGE analysis. (**a**) Workflow of protein spot selection and identification; (**b**) Statistical tests, group comparisons and number of selected spots; (**c**) Map of all identified variant spots in mouse serum following irradiation of the skin (spot numbers assigned by SameSpots). CTRL, sham-irradiated; IR, irradiated.

The data obtained with 2D-DIGE (see Supplemental Table S2 for the raw data of each spot at each dose and each time post-irradiation) were analyzed by univariate (ANOVA) and multivariate (PLS-DA and Random Forest) statistics to look for relevant proteins and for best candidates for triage and prognosis of radiation-induced skin burns. Figure 5a summarizes the workflow that led to the identification of proteins. ANOVA was able to highlight a number of protein candidates for assessing either dose or exposure. We selected a large set of interesting differentially expressed spots by comparing, at each time post-irradiation, (i) all controls (sham-irradiated) with all irradiated samples, whatever the dose of irradiation, and (ii) all irradiated samples between them, *i.e.*, 20 Gy *vs.* 40 Gy, 20 Gy *vs.* 80 Gy and 40 Gy *vs.* 80 Gy (Figure 5b). This strategy could allow us to highlight both spots expression of which was linked to ionizing radiation exposure and spots that varied with the dose of irradiation, which means spots varying with the severity of future lesions. A final number of 110 different spots, which took into account common differentially expressed spots (redundancies), were selected by this method. Since we had a reasonably large number of samples (16 per group), we then mined the data using supervised multivariate statistical analyses. The choice of the method was made after comparison of Random Forest and PLS-DA. The Random Forest method was given up because it provided less successful discriminations. This result was not very surprising given the relatively small sampling. The discriminating variables found by this method were, however, essentially the same as by the PLS-DA. The PLS-DA method is particularly well adapted to situations in which the number of variables is well above the number of individuals. At the end of every analysis the importance of every variable in the discrimination was supplied by the software. Figure 5b lists the number of variables, 17 in all, that were selected by PLS-DA for each comparison, taking into account common differentially expressed spots (redundancies). These candidates are shown and discussed below (see Section 3.3.4). Finally, a selection of 114 interesting spots was made. The corresponding assignment spot numbers of these spots are shown in the Figure 5c on a representative 2D-DIGE gel.

The 114 spots exhibiting significant expression changes were then submitted to identification using MS and database searches. By means of several MS methodologies, we managed to identify 86 of 114 spots. The results of MS identifications and the results of statistical tests performed to compare the levels of proteins between the different groups of individuals are given in Supplemental Table S3. A summary of the results of identification together with maximum fold changes and *p*-values, are presented in Table 1 where the 37 different proteins are reported. As described in our previous work, numerous spots were organized in spot trains of the same proteins. For instance, spots 407, 403, 400, 401, 404, 408, 412 were mostly identified as complement component 7, and spots 511, 512, 499, 509, 519, 541, 579, 578, 1681, identified as hemopexin, were organized in spot trains on 2D gels likely due to post-translational modification or proteolytic cleavages of the same proteins [18], which alter the electrophoretic mobilities of proteins, giving rise to distinct spots in 2D gels [37]. These post-translational modifications are possibly, at least in part, due to the glycosylation status of proteins. We showed using the same model that this status was modified after high doses of ionizing radiation [19]. 

In this study, we identified 37 distinct proteins that were differentially expressed after irradiation by comparing irradiated and sham-irradiated individuals, and by comparing all irradiated groups between them. Some of these proteins were previously identified in our first study [18]: serum protease inhibitor A3K (serpina3k), murinoglobulin, hemopexin, kininogen I, antithrombin III, α-1-anti-trypsin 1-1 and 1-2, haptoglobin, apolipoprotein A-I and peroxiredoxin 2. However, 26 additional proteins were highlighted by data analysis and were further identified in this study. There may be several reasons for this greater number of selected and identified proteins: (i) the use of more efficient software for image analysis, especially because the Progenesis Samespots software is quite effective in aligning and matching spots between gels; (ii) a large number of biological replicates that could make the statistics more powerful; (iii) the use of three doses of irradiation and two time points, while a single dose was used in the previous study; (iv) the way of analyzing the results, in particular by grouping the irradiated individuals and by using supervised and unsupervised multivariate statistics; and (v) the use of several mass spectrometry techniques that made identifications more successful.

#### 3.3.3. Pathway Analysis of Differentially Expressed Proteins Revealed by 2D-DIGE Analysis

**Figure 6:**
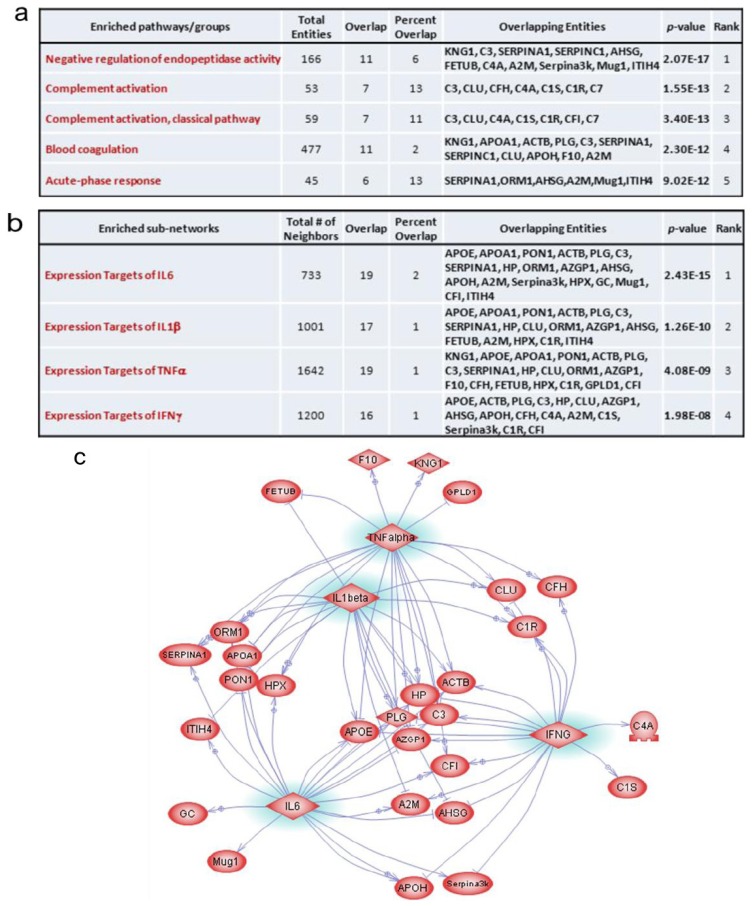
Pathway and sub-network enrichment and analysis. (**a**) Enriched pathway groups; (**b**) Enriched sub-networks; (**c**) Protein network of identified proteins linked by the common upstream regulators IL-1β, TNFα, IFNγ and IL-6.

The Pathway Studio software was employed to establish the molecular interaction and regulation network based on high-throughput interaction datasets. The presence of biological interactions of the 37 differentially regulated proteins was explored by interpreting high-throughput expression data to identify relationships among the proteins and cellular processes involved, and to organize the data into functional networks based on a repository database of known protein-protein interaction and functional interactions. Using the Gene Ontology (GO) enriched pathways/groups plugin of Pathway Studio, we first assessed whether there was any over-representation of GO categories in the networks. Significantly over-represented GO terms included those that are related to negative regulation of endopeptidase activity, complement activation, blood coagulation and acute phase response in the biological process-based network (Figure 6a).

**Table 1:** Differentially expressed spots post-irradiation identified in the study.

Protein name	Name in database	SwissProt ID	Number of spots ^1^	Spot number ^2^	Irradiation ^3,4^		Dose ^3,5^	
					Max fold change (*p* < 0.05)	*p*-value	Max fold change (*p* < 0.05)	*p*-value
**Actin, beta**	ACTB	ACTB_MOUSE	1	1668	=	/	−1.2	8.5 10^−3^
**Afamin**	AFM	AFAM_MOUSE	3	425, 438, 468	+1.2	5.4 10^−4^	+1.2	5.3 10^−3^
**Alpha-1-acid glycoprotein 1 (orosomucoid 1)**	ORM1	A1AG1_MOUSE	1	919	−1.2	3.7 10^−3^	=	/
**Alpha-1-antitrypsin 1-1**	SERPINA1	A1AT1_MOUSE	1	357	−1.2	2.0 10^−2^	−1.2	4.7 10^−2^
**Alpha-1-antitrypsin 1-2**	SERPINA1	A1AT2_MOUSE	1	683	−1.5	2.0 10^−3^	−1.4	2.4 10^−2^
**Alpha-2-HS-glycoprotein (Fetuin A)**	AHSG	FETUA_MOUSE	2	713, 849	−1.2	8.4 10^−4^	=	-
**Alpha-2-macroglobulin**	A2M	A2M_MOUSE	3	996, 1009, 1010	=	/	−1.5	5.4 10^−4^
**Antithrombin-III**	SERPINC1	ANT3_MOUSE	3	1695, 1697, 648	+1.2	1.2 10^−3^	+1.1	5.2 10^−3^
**Apolipoprotein A-I**	APOA1	APOA1_MOUSE	1	1335	=	/	−1.3	6.0 10^−4^
**Apolipoprotein E**	APOE	APOE_MOUSE	3	1080, 1099, 1106	−1.2	6.6 10^−3^	=	/
**Apolipoprotein H (Beta-2-glycoprotein I)**	APOH	APOH_MOUSE	1	626	+1.2	4.4 10^−3^	+1.2	8.2 10^−3^
**Clusterin**	CLU	CLUS_MOUSE	7	938, 1015, 1036, 1042, 1096, 1669, 1670	+1.3	2.8 10^−8^	+1.2	5.7 10^−7^
					−1.2	1.2 10^−3^	−1.2	2.2 10^−2^
**Coagulation factor X**	F10	FA10_MOUSE	1	778	−1.2	2.2 10^−3^	=	/
**Complement C1r-A subcomponent**	C1R	C1RA_MOUSE	2	475, 477	+1.2	2.3 10^−3^	+1.3	6.3 10^−5^
**Complement C1s-A subcomponent**	C1S	CS1A_MOUSE	1	476	+1.1	5 10^−3^	=	/
**Complement C3**	C3	CO3_MOUSE	4	579, 595, 1205, 1677	−1.3	2.9 10^−3^	−1.2	2.3 10^−3^
**Complement C4-B**	C4A	CO4B_MOUSE	1	1594	−1.3	1.7 10^−2^	=	/
**Complement component 7**	C7	CO7_HUMAN	5	400, 401, 407, 408, 412	−1.3	7.5 10^−4^	−1.4	2.9 10^−2^
**Complement factor H**	CFH	CFAH_MOUSE	1	164	−1.1	4.5 10^−3^	=	/
**Complement factor I**	CFI	CFAI_MOUSE	1	1057	+1.1	1.5 10^−3^	+1.2	2.0 10^−3^
**Fetuin B**	FETUB	FETUB_MOUSE	2	673, 676	+1.2	1.7 10^−5^	+1.1	2.4 10^−3^
**Group-specific component (Vitamin D binding protein)**	GC	VTDB_MOUSE	2	667, 678	+1.3	7.4 10^−4^	+1.3	4.3 10^−3^
**Haptoglobin**	HP	HPT_MOUSE	2	931, 942	+7.1	2.1 10^−7^	+2.5	4.6 10^−2^
**Hemopexin**	HPX	HEMO_MOUSE	9	499, 509, 511, 512, 519, 541, 578, 1681, 1692	−1.3	2.3 10^−3^	+1.2	6.9 10^−3^
					+1.3	4.5 10^−5^		
**Ig mu chain C region secreted form**	Ighm	IGHM_MOUSE	2	319, 358	−1.3	7.9 10^−3^	−1.1	2.0 10^−2^
**Inter alpha-trypsin inhibitor, heavy chain 4, isoform CRA_b**	ITIH4	A6X935_MOUSE	1	345	+1.1	1.0 10^−2^	+1.1	4.4 10^−2^
**Kininogen-1**	KNG1	KNG1_MOUSE	1	569	+1.3	1.2 10^−4^	=	/
**Murinoglobulin-1**	Mug1	MUG1_MOUSE	1	627	+1.1	2.5 10^−2^	+1.1	2.8 10^−3^
**Novel protein similar to odorant binding protein Ia Obp1a**	Gm5938	A2AEN9_MOUSE	1	1653	+1.5	1.1 10^−2^	=	/
**Odorant binding protein Ia**	Obp1a	P97336_MOUSE	1	1632	+1.6	4.2 10^-3^	=	/
**Pantothenate kinase 4**	PANK4	PANK4_MOUSE	1	1129	+1.9	2.9 10^-11^	+1.6	3.6 10^−7^
**Peroxiredoxin-2**	PRDX2	PRDX2_MOUSE	1	1413	=	/	−1.3	1.6 10^−2^
**Glycosylphosphatidylinositol specific phospholipase D1**	GPLD1	PHLD_MOUSE	2	355, 520	−1.3	8.0 10^-4^	−1.2	6.4 10^−3^
**Plasminogen**	PLG	PLMN_MOUSE	2	309, 415	−1.2	1.1 10^-3^	−1.7	4.2 10^−2^
					+1.6	7.9 10^-3^	+1.6	2.6 10^−2^
**Serine protease inhibitor A3K**	Serpina3k	SPA3K_MOUSE	12	54, 86, 87, 256, 257, 262, 531, 588, 599, 621, 1671, 1672	−1.6	2.0 10^-5^	−1.3	4.0 10^−2^
**Serum paraoxonase/ arylesterase 1**	PON1	PON1_MOUSE	1	803	−1.2	5.0 10^-3^	=	/
**Zinc-alpha-2-glycoprotein**	AZGP1	ZA2G_MOUSE	2	787, 790	+1.1	3.1 10^-2^	+1.1	4.1 10^−4^

^1^ Several differentially expressed spots were identified as same proteins; ^2^ Spot numbers were assigned by Progenesis SameSpots software according to their position in 2-DE gels; ^3^ Max Fold Changes associated with a *p*-value < 0.05. Fold changes and results of statistical tests (*p*- and *q*-values, Power) are comprehensively given in Supplemental Table S3. The different spots of Clusterin, Hemopexin and Plasminogen could display opposite trends of expression, which is why two different Max Fold Changes have been reported in the Table; ^4^ Max fold changes are given for irradiated samples compared to controls (sham-irradiated) samples; ^5^ Max fold changes are given for comparisons of irradiated samples (20, 40 and 80 Gy) (the highest dose *versus* the lowest dose).

Negative regulation of endopeptidase activity is any process that decreases the frequency, rate or extent of endopeptidase activity, the endohydrolysis of peptide bonds within proteins. This general process is involved in the protection of tissues against enzymes released by inflammatory cells. Given that radiation-induced inflammation and production of reactive oxygen species play a critical role in normal tissue response [38,39,40], this process was consistently found to be involved in the skin response to irradiation based on the proteomics data. The complement system is a set of plasma proteins that act together to attack extracellular forms of pathogens. Complement activation can occur spontaneously with certain pathogens or by antibody binding to the pathogen. Its early activation after irradiation may reflect a loss in the skin barrier, although no macroscopic sign was visible at this time. However, the interpretation of this information remains challenging since we do not have any functional data on the complement system in our model. The biological process of blood coagulation was also significantly over-represented. It has been extensively described that the vasculature is a critical target compartment involved in tissue response to radiation exposure and strongly participates in the initiation and development of radiation lesions [41,42]. As we previously observed, these variations may reflect the severe injury caused by irradiation to the vasculature [43], and may reveal an activation of the coagulation system in response to endothelial damage [18]. Lastly, the acute phase response was previously recognized as a major biological process in response to irradiation of the skin [18]. Acute phase proteins increase (positive APPs) or decrease (negative APPs) following different injuries, reflecting the occurrence and severity of inflammation [44]. Exposure to ionizing radiation has previously been shown to induce the production of APPs in several animal models [45,46,47,48,49] and during radiotherapy [50]. The changes in APPs of irradiated mice may serve as antioxidant and anti-inflammatory factors to reduce the damage to skin [49]. 

An interesting finding in this study is the identification of important protein networks around IL-1β, TNFα, IFNγ and IL-6 from pathway analysis using the enriched sub-networks plugin of Pathway Studio with which we searched for the significant common regulators (Figure 6b). We used these enriched sub-networks to build a pathway in which the selected sub-pathways were gathered. Based on this analysis, 28 of these 37 proteins were mapped onto this interacting network, showing the great importance of IL-1β, TNFα, IFNγ and IL-6 in the response to irradiation (Figure 6c). It is known that blood composition is modified after exposure to ionizing radiation through the immediate release by irradiated cells of cytokines and growth factors which stimulate neighboring cells or distant cells which in turn release proteins into the extracellular environment. IL-1, IL-6, IL-8, TGF-β, TNF-α and eotaxin are the major known cytokines involved in the response to ionizing radiation [19,51,52,53]. Cytokines play a role in mediating the inflammation process, and stimulate or repress APP synthesis in the liver [44], assisting the repair of the tissue. 

#### 3.3.4. Multivariate Statistical Analysis of 2D-DIGE Data: Best Candidates for Triage and Prognosis

**Figure 7:**
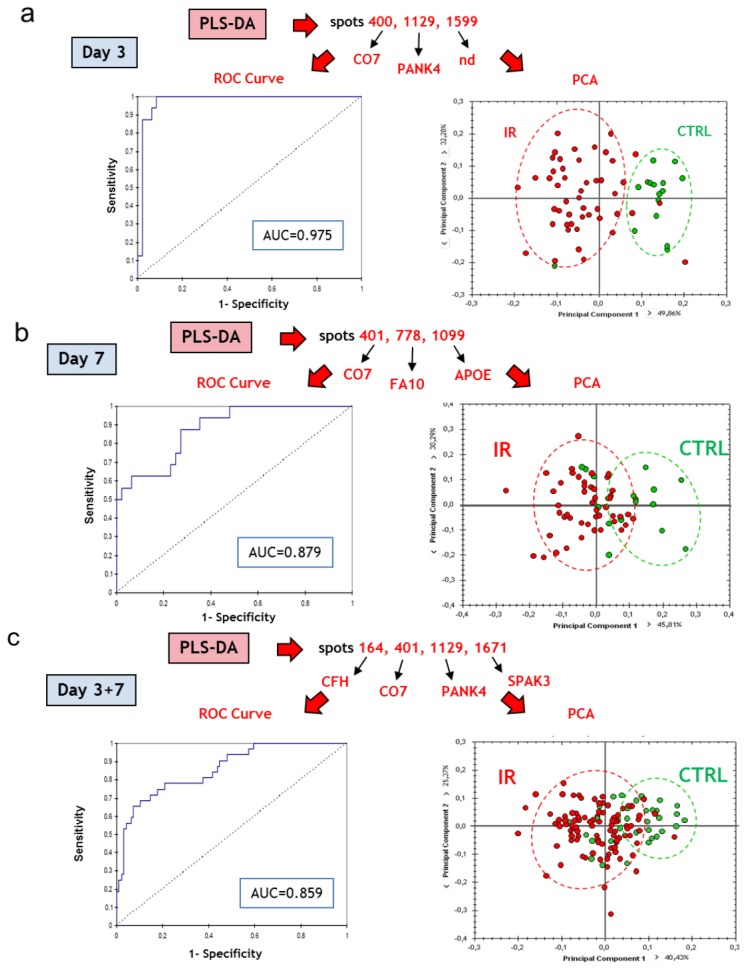
Top candidates for triage. (**a**) CTRL *vs.* all irradiated samples at day 3 post-irradiation; (**b**) CTRL *vs.* all irradiated samples at day 7 post-irradiation; (**c**) All CTRL *vs.* all irradiated samples at day 3 and day 7 post-irradiation. CTRL, sham-irradiated. IR, irradiated. Protein abbreviations are given in Table 1.

The best sets of candidates for the discrimination of groups proposed by the PLS-DA were used to plot ROC curves and calculate AUCs, and to perform PCA. The first question was to know whether it is possible to discriminate individuals according to their irradiation status, whatever the dose they received, *i.e.*, whether sets of serum proteins are suitable for early triage of locally exposed individuals. PLS-DA proposed different sets of three or four spots, depending on the day post-irradiation, which exhibit values of AUC above 0.85, showing that discriminations were satisfying (Figure 7a–c). These spots were identified by mass spectrometry (except for spot 1599).

We used the expression data of the spots selected by PLS-DA to perform PCA (Figure 7a–c). Interestingly, the discrimination was better at day 3 than at day 7, perhaps because increasing doses induced more differences in expression at later times, which reduces the importance of the difference between the control and the irradiated groups. In any case, we show here that the discrimination of locally irradiated individuals was possible using this methodology, even if the separation between irradiated and non-irradiated individuals was not perfect, especially when both times of blood collection post-irradiation were combined.

**Figure 8:**
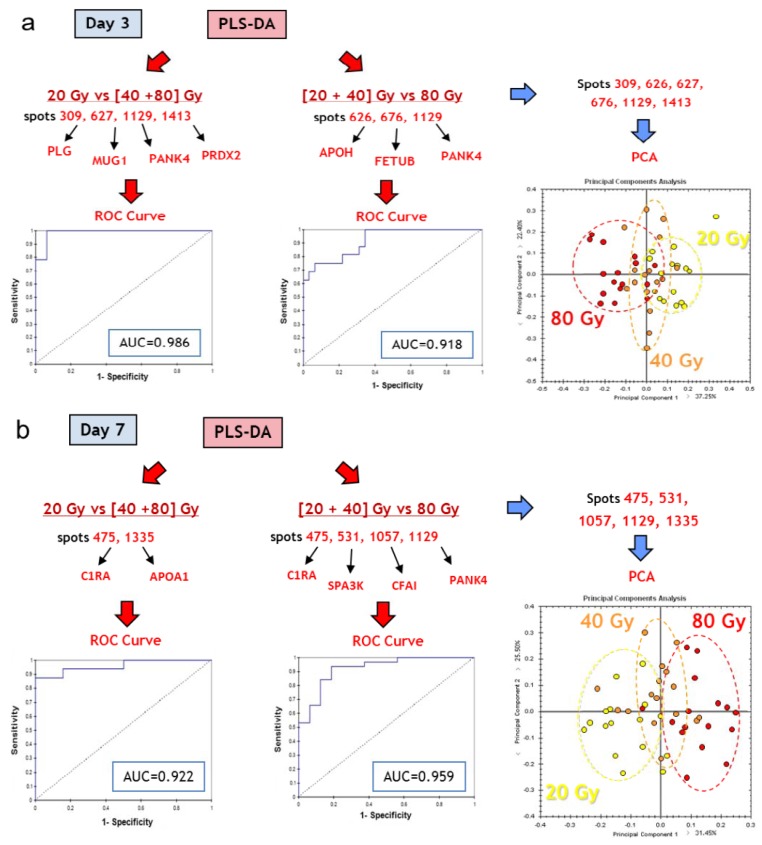
Top candidates for dose response. (**a**) Day 3 post-irradiation, 20 Gy *vs.* [40+80] Gy and [20+40] Gy *vs.* 80 Gy; (**b**) Day 7 post-irradiation, 20 Gy *vs.* [40+80] Gy and [20+40] Gy *vs.* 80 Gy. CTRL, sham-irradiated. IR, irradiated. Protein abbreviations are given in Table 1.

The second question was to know whether it is possible to discriminate early individuals that will develop lesions of different severities induced by different doses of ionizing radiation, *i.e.*, whether sets of serum proteins are able to predict the outcome of the lesion. To answer this question, we chose to compare groups of irradiated individuals. PLS-DA revealed that the discrimination of the group of 40 Gy was always poor, probably because this group is transitional between 20 and 80 Gy. To take into account this information, comparisons were either performed between the 20 Gy group and the 40+80 Gy group, or between the 20+40 Gy group and the 80 Gy group. PLS-DA proposed different sets of 2 or 4 spots, depending on the day post-irradiation, which displayed values of AUC above 0.90, showing that discriminations were satisfying (Figure 8a,b). These spots were identified by mass spectrometry. Their identities are given in Figure 8. For each day post-irradiation, all the spots highlighted by PLS-DA were used to perform PCA (Figure 8a,b). The results reflect that the 40 Gy group is intermediate between 20 and 80 Gy. The proposed sets of proteins may thus serve for the discrimination between uninjured/slightly injured animals and animals that developed severe lesions.

Based on PLS-DA, we propose 15 proteins, listed in Table 2, which constitute a set of candidate proteins for triage and prognosis of lesion outcomes. Table 3 reports the AUC for each set and each group comparison. Different combinations of these proteins should be tested in the course of a clinical trial to assess their potential to detect a localized irradiation or to predict the appearance of a severe lesion. This could be done, for instance, using samples from patients treated by radiotherapy for breast cancer in which the skin can be damaged during the treatment.

**Table 2:** Best candidates selected by PLS-DA for triage and prognosis of radiation-induced skin burns.

Best protein candidates based on PLS-DA							
Protein name	Accession (Uniprot KB)	Name in DB	Spot number ^1^	Irradiation ^2,3^		Dose ^2,4^	
				Max fold change (*p* < 0.05)	*p*-value	Max fold change (*p* < 0.05)	*p*-value
**Apolipoprotein A-I**	APOA1_MOUSE	APOA1	1335	=	/	−1.3	6.0 10^−4^
**Apolipoprotein E**	APOE_MOUSE	APOE	1099	−1.2	1.0 10^−2^	=	/
**Apolipoprotein H**	APOH_MOUSE	APOH	626	+1.2	4.4 10^−3^	+1.2	8.2 10^−3^
**Coagulation factor X**	FA10_MOUSE	F10	778	−1.2	2.2 10^−3^	=	/
**Complement C1r-A subcomponent**	C1RA_MOUSE	C1R	475	=	/	+1.3	6.3 10^−5^
**Complement component 7**	CO7_HUMAN	C7	400, 401	−1.3	2.5 10^−4^	=	/
**Complement factor H**	CFAH_MOUSE	CFH	164	+1.1	4.5 10^−3^	=	/
**Complement factor I**	CFAI_MOUSE	CFI	1057	−1.1	1.5 10^−3^	+1.2	2.0 10^−3^
**Fetuin B**	FETUB_MOUSE	FETUB	676	+1.2	1.7 10^−5^	+1.1	2.4 10^−3^
**Murinoglobulin 1**	MUG1_MOUSE	Mug1	627	+1.1	2.5 10^−2^	+1.1	2.8 10^−3^
**Pantothenate kinase 4**	PANK4_MOUSE	PANK4	1129	+1.9	2.9 10^−11^	+1.6	3.6 10^−7^
**Peroxiredoxin-2**	PRDX2_MOUSE	PRDX2	1413	=	-	−1.3	1.6 10^−2^
**Plasminogen**	PLMN_MOUSE	PLG	309	−1.2	1.1 10^−3^	−1.3	4.2 10^−3^
**Serine protease inhibitor A3K**	SPA3K_MOUSE	Serpina3k	1671	−1.6	2.0 10^−3^	−1.3	4.0 10^−2^
**Non-identified**	-	-	1599	+1.7	1.8 10^−3^	=	/

^1^ Spot numbers assigned by Progenesis SameSpots software according to their position in 2-DE gels; ^2^ Max Fold Changes associated with a *p*-value < 0.05. Fold changes and results of statistical tests (*p*- and *q*-values, Power) are comprehensively given in Supplemental Table S3; ^3^ Max fold changes are given for irradiated samples compared to controls (sham-irradiated) samples; ^4^ Max fold changes are given for comparisons of irradiated samples (20, 40 and 80 Gy) (the highest dose *versus* the lowest dose).

**Table 3:** AUCs associated with the comparisons of the different groups using the sets of proteins selected by PLS-DA.

Results for ROC curve analysis			
Discriminant variables	Group discrimination		AUC (ROC)
**C7/PANK4/spot 1599**	Irradiation	NI *vs*. IR, d3	0.975
**C7/F10/APOE**		NI *vs*. IR, d7	0.879
**C7/PANK4/Serpina3k/CFH**		NI *vs*. IR, d3+d7	0.859
**Mug1/PANK4/PRDX2/PLG**	Dose	20 Gy *vs*. (40+80) Gy, d3	0.986
**APOH/FETUB/PANK4**		(20+40) Gy *vs*. 80 Gy, d3	0.918
**C1RA/APOA1**		20 Gy *vs*. (40+80) Gy, d7	0.922
**C1RA/Serpina3k/CFI/PANK4**		(20+40) Gy *vs*. 80 Gy, d7	0.959

## 4. Conclusions

In this work, we investigated the power of proteomics to discriminate between non-irradiated and locally irradiated individuals, or between locally irradiated individuals that will develop lesion of different grades of severity. We exposed the skin of mice to high radiation doses of 20–80 Gy range in order to mimic two different exposure scenarios: exposure to a radiological source (by accident or caused by a terrorist act) and radiotherapy. These two scenarios can lead to exposure that will cause severe radiation burns if the radiation doses are greater than 20 Gy. Our work shows here that proteomics by 2D-DIGE-MS allowed the characterization of sets of proteins able to discriminate early between uninjured and injured animals. An interesting question is whether the results would be significantly different if later times were studied. This question merits further investigation. Conversely, SELDI-TOF-MS, which is an interesting methodology as regards its fast analysis and clinical capabilities, discriminated poorly between groups, likely because of low reliability of measurements. This is not surprising since SELDI-TOF-MS is a comparatively old technology, particularly when applied to a complex sample such as the serum proteome. SELDI-TOF is thus probably not suitable for triage or prediction of lesion severity. One can still keep in mind, however, that a more reliable and accurate high-throughput technology based on proteomic profiling can be used as a mobile apparatus in case of radiological accident. On the other hand, 2D-DIGE remains a powerful and promising method for the discovery of sets of proteins that could be used for the development of clinical tests for the triage and/or prediction of radiation-induced skin lesion severities. Nevertheless, identifications and validations are not trivial, in particular because post-translational modifications can be responsible for quantitative variations of 2D-DIGE spots, as previously shown [19]. In this work, we propose a set of 15 proteins based on 2D-DIGE analyses that could be useful for diagnosis and prediction of radiation burns. These proteins should now be investigated in human subjects treated by radiotherapy to check if their expressions are modified following radiation exposure. Another important study should focus on the ability to detect glycosylation modifications of serum proteins, since these modifications may also signal or predict a radiation burn [19]. Finally, rather than the consideration of only a few proteins, future prospects in radiation research will rather have to connect molecular biology to radiation pathophysiology and radiation disease through molecular networks of different origins: DNA (SNPs), gene expression, protein expression, metabolite expression, *etc*. [15,17]. Variations of this network can, in turn, be considered as a sensor of a disease state as that caused by ionizing radiation. These molecular networks will likely be essential to study, detect and predict radiation effects.

## Supplementary Materials

Supplementary File 1

Supplementary File 2

Supplementary File 3
